# Anticipatory and reactive mechanisms of habituation to visual distractors

**DOI:** 10.1038/s41598-025-04082-5

**Published:** 2025-07-02

**Authors:** Bram Burleson, Massimo Turatto, Gijs Plomp, David Pascucci

**Affiliations:** 1 Department of Basic Neurosciences, Faculty of Medecine, University of Geneva, Geneva, Switzerland; 2 Center for Mind/Brain Sciences (CIMeC), University of Trento, Rovereto, Italy; 3 Perceptual Networks Group, Department of Psychology, University of Fribourg, Fribourg, Switzerland; 4 Laboratory of Psychophysics, Brain Mind Institute, École Polytechnique Fédérale de Lausanne (EPFL), Lausanne, Switzerland; 5 The Radiology Department, Lausanne University Hospital and University of Lausanne, Lausanne, Switzerland; 6 The Sense Innovation and Research Center, Lausanne and Sion, Switzerland

**Keywords:** Attention, Distractors, Habituation, Alpha rhythms, EEG, Human behaviour, Attention, Habituation, Electroencephalography - EEG

## Abstract

Human attention can rapidly habituate to irrelevant and repetitive visual distractors. Although this phenomenon is well-documented in behavioral studies, the neural mechanisms involved remain largely unknown. In this study, we investigated the neural correlates of attentional habituation using scalp electroencephalography (EEG). Participants performed a visual discrimination task while intermittently presented with salient distractor stimuli. The cost in reaction times (RT) associated with the distractor exhibited the typical time course of habituation, decreasing as a function of repeated exposure to the distractor. We found that this habituation coincided with both reactive and proactive changes in EEG activity. Post-distractor reactive EEG components emerged gradually over the course of the experiment, likely reflecting the operation of an inhibitory network aimed at suppressing distractor interference in the main task. Pre-stimulus α rhythms gradually tuned their power peaks to the anticipated moment of the distractor, suggesting the involvement of predictive inhibitory models based on prior experience with the distractor. Collectively, our findings suggest that attentional habituation involves multi-stage interacting mechanisms that anticipate the occurrence of a distractor and facilitate the rapid reallocation of attentional resources away from the distractor.

## Introduction

The ability to disregard repetitive yet meaningless stimuli is a common trait across species. From reflexive responses in simple organisms to cognitive processes in complex animals, reactions to inconsequential stimuli decrease rapidly upon repeated exposure, a phenomenon known as *habituation*^1,2^.

**Fig. 1:**
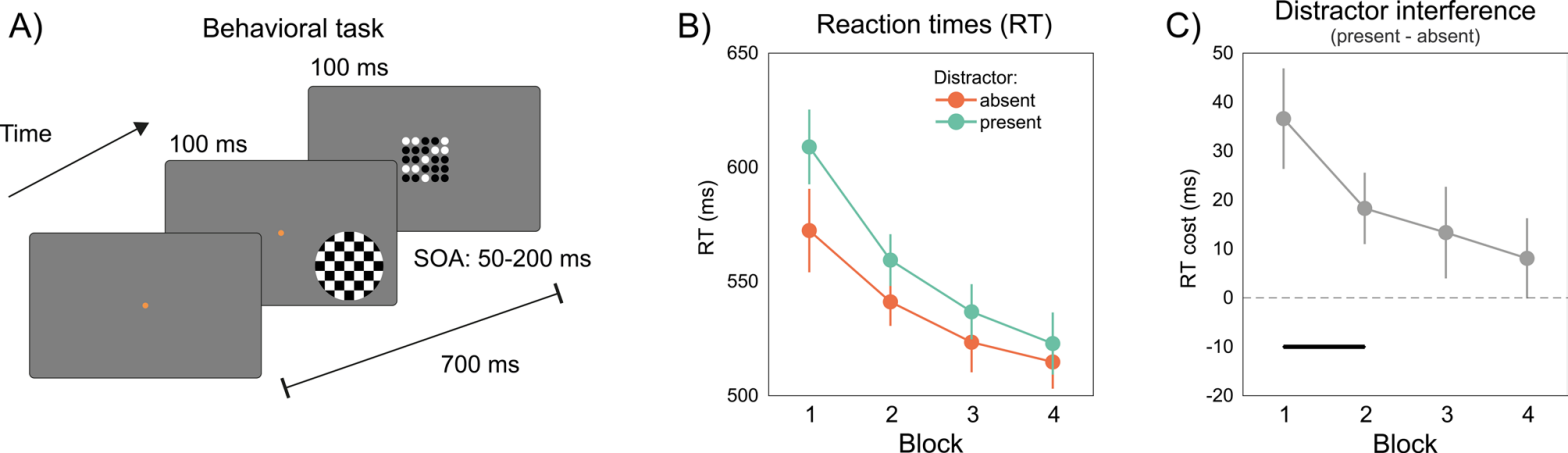
Habituation to visual distractors: task and behavioral results. (**A**) The sequence of events in a single trial with a distractor present (stimuli are not drawn to scale). The distractor, a black and white checkerboard, appeared in 40% of the trials either to the left or right of fixation. In trials with a distractor present, the distractor was followed by a central matrix of dots. Participants were asked to report whether the central matrix, the target of the visual task, contained more white or black dots. In trials without a distractor, the structure and temporal sequence of events remained the same, but the distractor was not presented. (**B**) RTs in the conditions with the distractor absent (orange dots and lines) and present (green dots and lines), averaged across participants over the four blocks of the experiment. (**C**) Measure of distractor interference, computed as the difference between RT in trials with the distractor present and those without it (i.e., quantifying the RT cost due to the presence of a distractor, with higher RT costs indicating greater interference caused by the distractor). The horizontal gray line highlights blocks where distractor interference was significantly larger than 0 (see “Results”). Error bars indicate 95% CI^84^.

In humans, habituation is evident in a variety of elementary physiological responses, including skin conductance responses^3^, heart rate^4^, pupil dilation^5^, and event-related scalp potentials^6,7^. Habituation can also affect more complex and hidden aspects of human behavior, such as the attentional and oculomotor responses after repeated exposure to an irrelevant visual distractor^8–11^. In a typical paradigm, participants engage in a visual task while an irrelevant but attention-grabbing stimulus called ‘distractor’ occasionally occurs (Fig. 1). Likely due to an involuntary shift of attention, the distractor initially slows down reaction times (RTs) in the primary task, causing a cost in RTs when compared to trials without a distractor —i.e., an effect of attentional capture. Such distractor interference decreases rapidly across blocks of trials^11^. Strikingly, habituation of the distractor interference manifests even when participants are not engaged in any explicit visual discrimination task—i.e., during passive viewing^10^—and once established, can endure for days and weeks^10,11^.

Classic theories of habituation, like the *stimulus-model comparator* proposed by Sokolov^12^, postulate that the brain forms a neural model of the irrelevant stimulus based on its history of occurrence, and this model is used to make predictions about future events, against which the current sensory input is compared^13^. In this perspective, an ancestor of contemporary predictive coding frameworks^14,15^, once the brain has learned the features of a repetitive distractor, the more a new stimulus matches the distractor, the more habituated the elicited response becomes. In line with this theoretical framework, it has been suggested that habituation relies on stored representations, or ‘inhibitory templates’^16–18^, based on the expected frequency and contextual properties of distractors^9^.

Importantly, models of habituation conceptualize this phenomenon as a stable and enduring form of learning that transcends simple inter-trial adaptation or repetition effects. Unlike transient effects limited to the immediately preceding trial, indeed, habituation involves long-lasting changes that depend on the learning of statistical and contextual regularities. These regularities include not only the frequency of a stimulus but also its spatiotemporal and contextual properties, such as the likelihood of its occurrence at specific locations, times, or in particular contexts^10,11,19–21^. Thus, both traditional and recent theories propose that habituation involves two primary processes: (1) generating predictions about upcoming events from an internal model based on the prior history of stimulation, and (2) mechanisms that suppress the response elicited by the sensory input when this matches the prediction. In the context of habituation to visual distractors, however, how these two processes are implemented is not yet understood.

Two candidate mechanisms that may support the processes involved in habituation are those that operate in reaction to a distractor^22–26^ and those that operate in anticipation of a distractor^27,28^. Both have been widely documented, each associated with distinctive neural signatures. These mechanisms can be loosely termed reactive and proactive, respectively, based on their temporal relationship to the stimulus and their role in either suppressing responses to distractors or anticipating and preventing distraction in advance^29^. Although these terms may carry different connotations under various theoretical perspectives, we will use them in a purely descriptive manner throughout the manuscript to refer to post-stimulus (reactive) and pre-stimulus (proactive) components.

Reactive suppression has been related to broad (100–400 ms) changes in electroencephalography (EEG) evoked potentials^22–24,26^. Conversely, proactive mechanisms may operate through cortical oscillations in the broad alpha range (α; 7–14 Hz)^27,28^, which facilitate pulsed inhibition and gating of sensory activity in anticipation of expected distractors^25,30–32^. While these components have been linked to distractor processing in a variety of classic paradigms of attention, their role and contribution to the establishment of inhibitory templates for habituation remains unclear.

Here, we investigated the EEG correlates of habituation to visual onset distractors. We focused on proactive and reactive changes in EEG activity triggered by a salient and repetitive distractor over time, which can be specifically linked to the build-up of habituation processes. During the experiment, participants engaged in a visual task at fixation, while a salient checkerboard visual distractor was occasionally presented (in 40% of the trials) at two possible peripheral locations, either on the left or right side. As the experiment progressed, the initially robust interference elicited by the distractor diminished, with RTs eventually approximating those in trials without the distractor, thus documenting an almost complete filtering of the irrelevant visual onset (see also^11^).

In line with the two main processes posited by classic models of habituation^12^, we found that habituation to visual distractors coincided with changes in both reactive and proactive EEG components. The former involved changes in evoked post-stimulus EEG topographies starting from approximately 160 ms after the distractor presentation. The latter involved the temporal tuning of pre-stimulus α power effects around the onset of the distractor: peaks in alpha power coinciding with the expected time window of the distractor led to reduced distractor interference as attested by faster RTs.

These findings are consistent with the idea of a multi-stage process involved in habituation and suggest a possible gradual build-up of inhibitory models^16,18,33^. Anticipatory mechanisms may adaptively inhibit the distractor based on its expected properties, while reactive modulations of attentional processing could help mitigate any residual distractor processing. We propose that these initial results serve as a starting ground and framework for future research to assess the characteristics and neural correlates of attentional habituation across diverse paradigms and conditions.

## Results

We investigated the EEG correlates associated with habituation to visual distractors during a visual attention task. Participants were presented with a series of trials, requiring them to determine whether a grid of dots at the center of the screen contained more black or white dots. In 40% of the trials, a salient distractor (a circular checkerboard stimulus, see Fig. 1A) was presented before the target grid. Our analyses focused on four key aspects: (1) the behavioral manifestation of habituation, attested by the decrement in RTs cost, or distractor interference, following repeated exposure to the distractor; (2) the correlates of habituation in post-stimulus event-related potentials (ERPs); (3) the correlates of habituation in pre-stimulus EEG oscillations, and; (4) the predictive relationships between EEG correlates of habituation and behavior. Herein, we present the findings from each of these analytical steps.

### Habituation to visual distractors

**Fig. 2:**
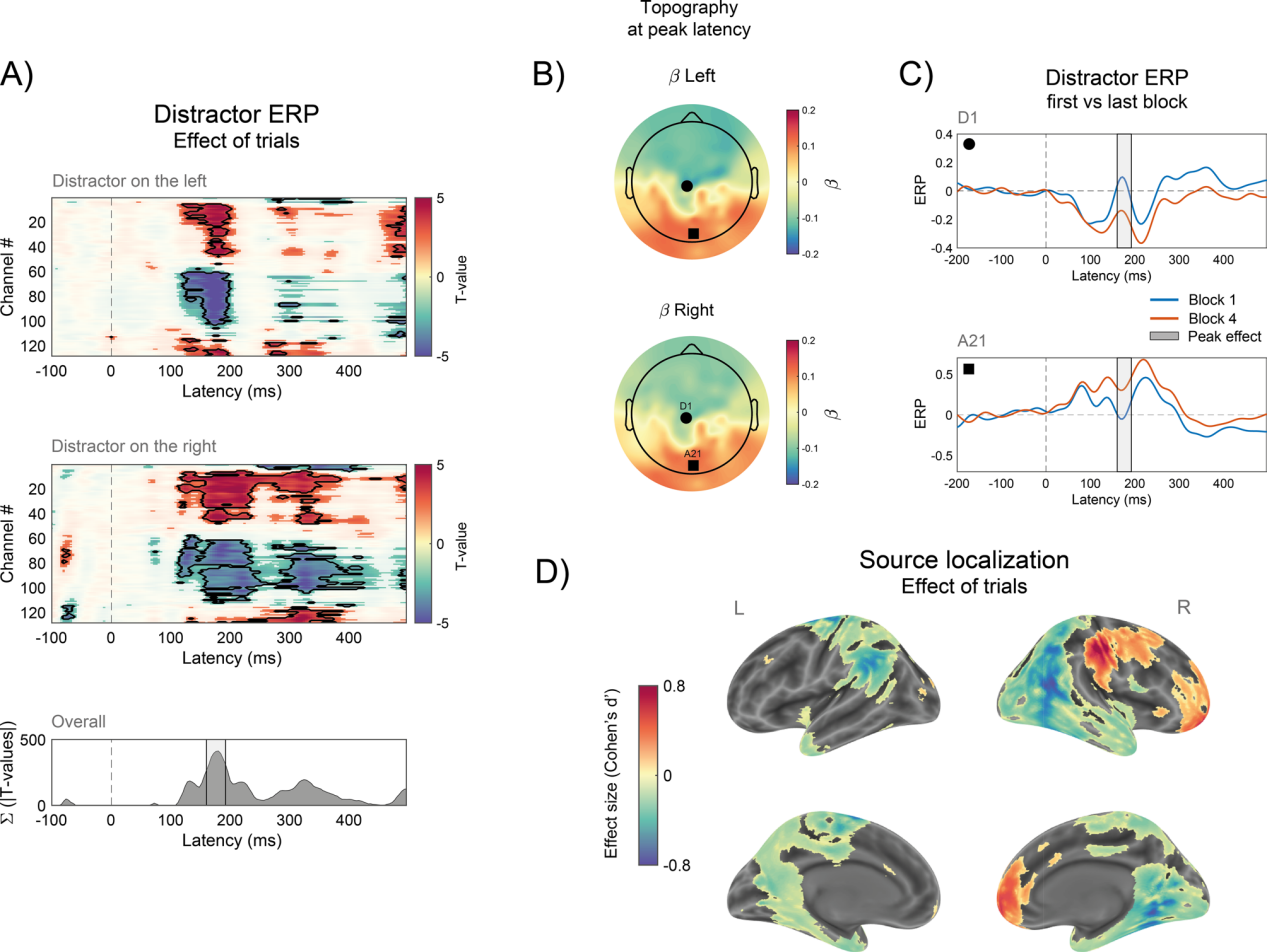
ERP correlates of habituation. (**A**) Habituation effects on distractor-ERP across all 128 channels, presented as a T-value heat map. Opaque regions indicate a significant effect of trials at α(uncorrected) = 0.05, while areas outlined in black denote significant effects after correction for multiple comparisons (see “Methods” and “Results”). The first and second heat plot show the results separately for conditions in which the distractor was presented on the left (top) and right (bottom). The lower panel shows the sum of T-values across significant electrodes and distractor location conditions, over time (after correction for multiple comparison, see main text). (**B**) Scalp topographies of coefficients estimated at the peak latency of the trial effect (160–192 ms), averaged across participants. (**C**) ERP at the two electrodes exhibiting the largest positive and negative coefficients of the trial effect, displayed separately for the first and last block of trials. (**D**) Source localization results of the effect of trials (effect size, see “Methods”, “Results” and Supplementary Table 1).

Throughout the experiment, performance improved in the central task, with a gradual decrease in RTs (Fig. 2 left). Distractor interference, as indexed by the RT cost in the presence of a distractor, was initially strong, but decreased rapidly over the four blocks, revealing the typical pattern of habituation (Fig. 1B,C). A two-way repeated-measures ANOVA with Distractor (present vs. absent) and Block (1–4) as factors, revealed a significant main effect of Distractor (F(1, 17) = 32.52, *p* < 0.001, ${\eta }_{p}^{2}$ = 0.65) and Block (F(3, 51) = 26.83, *p* < 0.001, ${\eta }_{p}^{2}$ = 0.61), and a significant interaction (F(3, 51) = 8.75, *p* < 0.001, ${\eta }_{p}^{2}$ = 0.34). Interference was strong in the first two blocks (block 1: t(17) = − 6.91, *p* < 0.001, Cohen’s *d’* = − 1.62; block 2: t(17) = − 4.77, *p* < 0.001, *d’* = − 1.12) but absent in the second half of the experiment (block 3: t(17) = − 2.60, *p* = 0.018, Cohen’s *d’* = − 0.61; block 4: t(17) = − 1.51, *p* = 0.148, *d’* = − 0.35; post hoc testing, Bonferroni correction with corrected alpha of 0.0125).

### ERP correlates of habituation

Habituation may lead to changes in post-distractor ERPs, reflecting differential processing of the distractor over time. Previous research on ERP and distractor suppressions has identified components like the distractor positivity (P_D_^24^) that emerge on average—i.e., across all trials, when contrasting ERPs contralateral or ipsilateral to a distractor, or ERPs for salient and non-salient distractors. However, the dynamics of ERPs during habituation to visual distractors remain unexplored. Here, we employed a data-driven approach^34^ to investigate changes in distractor-related ERPs across the entire scalp (see “Methods”).

We modeled single-electrode ERPs, time-locked to the distractor, as a function of the increasing number of trials, separating conditions where the distractor appeared on the left or right side. The results revealed a significant effect of ERP, broadly distributed across the scalp and emerging approximately 100 ms from the distractor (Fig. 2A). This effect was largely consistent between distractor sides, indicating a non-lateralized ERP component (see also Supplementary Material, Figure S1). Considering all significant effects, regardless of the side, the distribution of effect sizes exhibited a distinct peak in the 160–192 ms post-distractor time window. Within this time frame, a clear change in the overall topography emerged with repeated exposure to the distractor, indicating effects not confined to a single electrode. Notably, the results showed a marked increase in negative potentials over the frontal region and positive potentials over the occipital region (Fig. 2B).

Closer examination of the ERPs at the two electrodes displaying the most pronounced negative and positive effects, (A21 and D1, corresponding approximately to POz and CZ in the standard 10–20 system, respectively) showed evidence of modulation in relatively early ERP components (Fig. 2C), corresponding to the typical N1 and P2 visual ERP time windows^35^. A more traditional ERP analysis, focusing on changes in contra- and ipsilateral pre-selected electrodes, confirmed a distributed—i.e., non-lateralized—modulation of ERP amplitudes with repeated exposure to the distractor (see Supplementary Material and Figure S2).

To determine if the observed ERP modulations were related to behavioral responses, specifically RTs in distractor-present trials, and not merely due to time-on-task effects, we conducted an additional analysis. We used a linear model to predict log-transformed RTs based on ERP amplitudes averaged within the 160–192 ms window at the representative occipital electrode A21. The model yielded a significant intercept (6.65, SE = 0.047, t(3753) = 142.08, *p* < 0.001), a significant effect of log-trial (slope = − 0.064, SE = 0.0069, t(3753) = − 9.30, *p* < 0.001), and a significant negative effect of ERP amplitude (slope = − 0.0086, SE = 0.0033, t(3753) = − 2.63, *p* = 0.008). These results indicate that larger ERP amplitudes were associated with faster RTs. Thus, after accounting for time-on-task effects, the ERP modulations observed throughout the experiment were predictive of RTs in distractor-present trials, suggesting a direct relationship between these ERP changes and the suppression of distractor interference.

We then used distributed EEG source reconstruction to identify cortical regions underlying the observed changes in topographies over time. Estimates of single-trial source activity within the 160–192 ms time window were obtained using LCMV-beamforming (see “EEG source imaging”). Following the modeling approach used for ERPs, we fitted the time course of cortical source activity as a function of the increasing number of trials (here divided into 12 sub-blocks, see “Methods”). For each cortical source, we then estimated the t-value associated with the slope of the effect of sub-blocks at the single-subject level (positive meaning activity that increases over time, negative meaning decreases) and computed Cohen’s *d’* as a measure of the effect size at the group level, resulting in a distribution of effect sizes across the entire cortical surface. The results indicated an increase in activity in mid-frontal regions and a decrease in activity in more parietal and occipital regions. Specifically, over the course of the experiment and in parallel with the development of behavioral habituation, EEG activity in the 160–192 ms time window was consistent with an increase in regions including the right middle and superior frontal gyrus, and a broader decrease in activity in more posterior regions, including regions in the fusiform, lingual and temporal gyrus (see Fig. 1D and the table in Supplementary Material).

### Changes in pre-stimulus brain rhythms

With repeated exposure to the visual distractor, we found both an attentional habituation, and a progressive change in post-stimulus EEG topographies. Here, we explored the effect of time (trial number) on the power of pre-stimulus brain rhythms, which have been extensively linked to anticipatory mechanisms for distractor suppression.

**Fig. 3:**
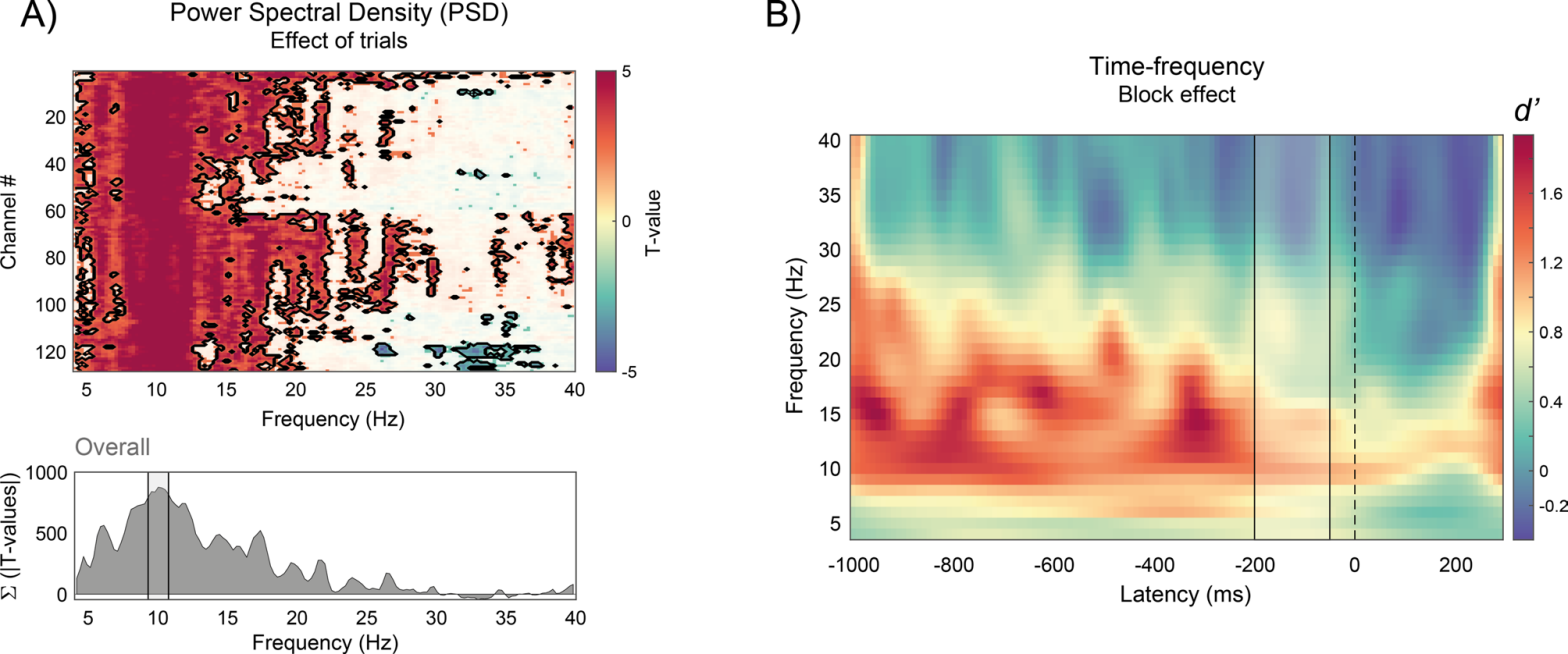
Changes in pre-stimulus brain rhythms. (**A**) Effect of trials on Power Spectral Density (PSD) in the 4–40 Hz range, across all 128 channels, presented as a heat map. Opaque regions indicate a significant effect of trials at α(uncorrected) = 0.05, while areas outlined in black denote significant effects after correction for multiple comparisons (dark red indicates power increases, dark blue indicates power decreases; see “Methods” and “Results”). The lower panel shows the sum of T-values across significant electrodes. The shaded gray rectangle highlights the frequency band with the strongest effect of trials (see “Results”). (**B**) Time–frequency plot showing the effect of block (last two blocks minus first two blocks, effect size reported as Cohen’s d’) on the power of EEG rhythms in the 4–40 Hz. The grey rectangle highlights the moments in which a distractor could appear, whereas the dashed vertical line indicates the onset of the target.

We employed a similar modeling approach as used for ERPs, but this time to investigate changes in pre-stimulus (from − 700 to 100 ms) power spectral density (PSD; within the 4–40 Hz range) over the course of the experiment. The results from the linear model (see “Pre-stimulus spectral analysis”) revealed a global increase in PSD across all electrodes. This effect was most pronounced in the α frequency band, specifically between 9–11 Hz (9.27–10.74 Hz; Fig. 3A). Further supporting this finding, a time–frequency analysis of pre-stimulus oscillatory dynamics in the 4–40 Hz frequency range, time-locked to the target onset, revealed a substantial increase in pre-stimulus α and low β activity in the last two blocks—where habituation has largely already occurred—compared to the initial two blocks—where habituation was starting to develop in behavior. Notably, only α effects extended into the time window of an expected distractor (Fig. 3B).

### Anticipatory α power modulations predict RT

Changes in α power throughout an experiment are not surprising and not necessarily a signature of habituation to the visual distractor. Indeed, they may well reflect task automaticity^36^ or fatigue effects^37^, in line with the classic ‘idling’ view of α activity^38^. Such effects, however, may not necessarily be related to processes involved in habituation to the visual distractor.

To assess the specific involvement of pre-stimulus α power modulations in habituation (i.e. in relation to the distractor processing), we ran a time-resolved linear mixed model. This model predicted log-transformed RT based on the scalp power of pre-stimulus α activity (9–11 Hz) and the interaction between pre-stimulus α power and distractor condition. The analysis, performed after adjusting for the effect of distractor condition on RT and the common trial effect (i.e., time-on-task effect) on both α power and RT, revealed distinct effects of α power.

**Fig. 4:**
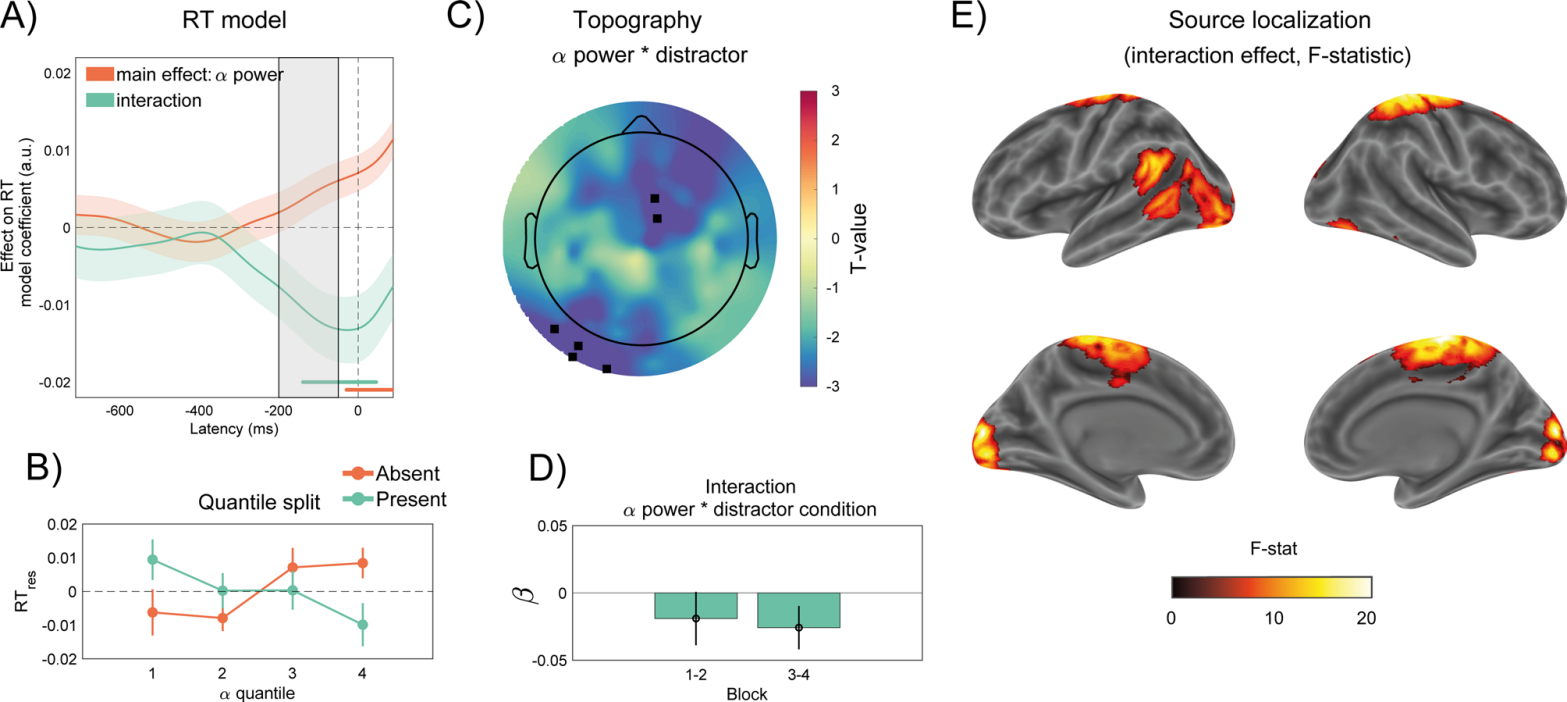
Anticipatory α power modulations predict RT (see Eqs. 2–4). (**A**) The orange line and shaded intervals show the main effect of α power on RTs, while the green line and shaded intervals represent the interaction between α power and the distractor condition on RTs, based on a pre-stimulus time-sliding regression model. This model accounts for time-on-task effects on both RT and α power (see “Methods” and “Results” for details on variable transformations). The grey rectangle marks the time window during which a distractor could appear, and the dashed vertical line indicates the target onset. Horizontal lines below the plot show time points where effects were statistically significant after false discovery rate (FDR) correction, with colors corresponding to their respective conditions. The results demonstrate that although higher α power during the target presentation generally slows down RTs, in distractor-present trials, an increase in α power during the distractor presentation reverses this effect. (**B**) Quantile plot of the α power effect on RTs (after accounting for time-on-task effects) in distractor-present (green) and distractor-absent (orange) trials. Error bars represent standard error of the mean (SEM). This plot highlights the pattern of the interaction shown in (**A**), where RTs increase with α power in distractor-absent trials but decrease in distractor-present trials. (**C**) Topography illustrating the interaction effect (T-values) at the significant time window highlighted by the green vertical bar in (**A**). (**D**) Coefficients and 95% confidence intervals of the interaction effect, assessed separately for the first and second halves of the experiment (Blocks 1–2 and 3–4). (**E**) Source localization results for the interaction effect described in (**A**), presented as thresholded F-statistics (refer to “Methods”, “Results” and Supplementary Table 1 for details).

First, in distractor-absent trials, increased α power in the late pre-stimulus interval coinciding with the onset of the central target led to a slowing down of RT (Fig. 4A, orange line). Second, in distractor-present trials the effect of increasing α power was reversed, resulting in faster RT (Fig. 4A, green line). This interaction effect, significant around the distractor onset and beyond, suggests that when α power increased during the distractor moment, the cost in RT due to the distractor (i.e., distractor interference) was reduced. During this time window, RTs were slower when α power was high in distractor-absent trials and faster when α power was high in distractor-present trials (Fig. 4B). This effect was most pronounced at occipital and mid-frontal electrode sites (Fig. 4C).

The analysis suggests that pre-stimulus α power modulations have differential effects on RT, contingent on the presence of a distractor. Crucially, the effect observed in distractor-present trials appears to be driven by α rhythms involved in diminishing distractor interference, a process potentially linked to habituation. To further investigate whether this effect was evident throughout the entire experiment or was more restricted to specific moments during the development of habituation, additional linear models were performed. These models, focusing on the time window where the interaction between α power and the distractor was significant, were separately conducted for each block. The results indicated that the interaction effect was more evident after the initial blocks (Fig. 4D), coinciding with the time when habituation to visual distractors was evident in behavior (Fig. 1B,C). Source localization revealed that the interaction effect found in pre-stimulus α-band activity (Fig. 4A) was consistent with power changes localized in both early visual areas and frontal regions including the superior frontal gyrus (Fig. 4E, see also the table in Supplementary Material).

Importantly, α power changes in this pre-stimulus time window also predicted the magnitude of the ERP associated with habituation in response to the distractor across trials (linear model predicting ERP with pre-stimulus α power in distractor present trials, slope of the effect of α = 0.126 ± 0.025, t = 4.95, *p* < 0.001, see “Methods”).

## Discussion

In this study, we investigated the neural correlates of habituation to visual distractors. To this aim, we focused on EEG scalp activity changes both in reaction to and in anticipation of a repetitive salient visual onset distractor.

We found that the strength of distractor interference, as indexed by the RT cost induced by a salient distractor, decreased rapidly within a few tens of trials. This time course closely resembles the temporal dynamics reported in previous studies on habituation in visual attention, despite differences in paradigms and stimuli^8–11,21,39–42^, suggesting that the phenomenon we observed is both general and reliable. These findings, along with a substantial body of research on statistical learning effects on distractor suppression^16,29,43–45^, highlight the critical role of prior experience in learning to suppress visual distractors. Moreover, the clear and robust initial RT cost caused by the distractor—i.e., attentional capture—confirms the well-established finding that onsets and visual transients reliably capture attention^8–11,41,46,47^, challenging the notion that an onset appearing outside the monitored attentional “window” should not^48^.

These effects cannot be fully explained by task learning or increased automaticity. One might argue that RTs in both distractor-present and distractor-absent trials decrease due to task learning, and that the interaction we observed between distractor condition and trial block reflects a floor effect in distractor-absent trials, where RTs plateau in the absence of distractors. While this remains a possibility in our study, future research with more targeted manipulations (e.g., omitting the distractor for several trials or presenting a novel distractor with different features) should address this more directly. However, based on previous studies employing very similar designs, this explanation conflicts with key characteristics of habituation, which extend beyond general time-on-task effects. For example, it has been shown that the reduction in the RT cost due to a distractor can emerge purely through passive exposure to the distractor, without active task engagement and thus, no task-learning effect^10^. Additionally, habituation effects are shaped by contextual factors^19,20^ and by prolonged experience with the distractor beyond a single-session learning effect^9^. Taken together, this literature indicates that habituation effects, like those reported here, cannot be attributed solely to general learning or floor effects.

In line with the stages of anticipation and reaction proposed by Sokolov’s 'stimulus-model comparator’ theory^12^, our EEG analyses revealed candidate correlates of habituation to visual onsets involving both pre- and post-distractor activity. This suggests that habituation mechanisms might operate through sequential, reactive and proactive attentional stages^29^. Reactive components, traditionally associated with suppressive mechanisms in distractor processing, aim at downregulating the impact and processing time of a distractor^16,49–51^. For instance, the P_D_ component, a lateralized ERP, often observed within a broad post-distractor time window (e.g., from 100 to 400 ms), has been proposed to index the degree of suppression following initial interference due to a distractor^23,51^. Similarly, the Selection Negativity (SN), a non-lateralized component evident over posterior electrode sites starting from ~ 150 ms, is supposed to downregulate the processing of an irrelevant stimulus presented even in the absence of a relevant target^22,25^. The latencies and phenomenology of these components indicate mechanisms for the reactive handling of salient visual distractors that may counteract the initial automatic interference they provoke, reducing the time spent on the distractor and the amount of processing resources engaged^23,52,53^.

In our study, we identified a non-lateralized modulation of ERP amplitude within a relatively early post-distractor time window (160 ms to 192 ms), which unfolded during habituation. This was characterized by increasing negative polarity over frontal electrodes and increasing positive polarity over occipital electrodes, around the time of classic attention-related ERP components N1 and P2^35^. We must note that we found no reliable effects of habituation in earlier, purely stimulus-driven ERPs (see also Supplementary Material and Figure S1). Thus, unlike habituation in other domains, which often involves attenuation of responses at early sensory processing stages, our results suggest a habituation process operating at later processing stages.

The observed modulations and the P_D_-SN components may reflect a common underlying mechanism, aimed at reactively suppressing and rapidly disengaging attention from distractors, which becomes more efficient over time and with repeated exposure to the same distractor^10^. These mechanisms appear as a plausible correlates of the reactive stage of habituation, as posited in the 'stimulus-model comparator’ theory^12^, suppressing the response to stimuli that match the predictions of an internal model.

However, we must note that we cannot directly link the distributed scalp effects observed here to the typical P_D_ or SN components, due to differences in display setups and analytical approaches. For example, P_D_ is usually identified using multi-element symmetric displays by subtracting ERPs contralateral to the distractor from those ipsilateral. This classic lateralized analysis may not reveal the same ERP shape when only a single distractor is presented peripherally (see Supplementary Material and Figure S2). Future studies incorporating habituation paradigms with multi-element displays could help to further characterize these effects and establish more direct links to classic ERP components, such as the PD.

A recent study^54^ identified an early positive deflection over ipsilateral electrodes, referred to as the ipsilateral visual orienting activity (VOA), which is associated with the covert orienting of attention toward a peripheral stimulus. It would be reasonable to expect habituation to modulate this early ipsilateral component^55^. For instance, during the initial phase of habituation, one might expect a pronounced VOA reflecting the orienting of attention toward the distractor that diminishes as the orienting response habituates. However, a dedicated analysis of lateralized electrodes (PO7/PO8 as in^54^), revealed no evidence of a VOA-like ERP either during the initial blocks or a modulation across trial blocks. Instead, we observed a bilateral amplitude increase as a function of trial block (see Supplementary Material and Figure S2). This pattern aligns with the time windows and distributed scalp effects observed in our main analysis (see “ERP correlates of habituation”).

The absence of a VOA-like ERP in our data remains difficult to interpret. One possibility is that it reflects differences in task demands. In McDonald et al.^54^, the VOA was observed when attention was voluntarily directed to the periphery rather than the center. Both peripheral and central stimuli were always shown. In contrast, in our task, peripheral onsets were task-irrelevant distractors that appeared only on 40% of the trials, and participants were explicitly instructed to ignore these distractors while maintaining their attention on the central stimulus. Thus, the VOA might be triggered by the voluntary orienting of attention to the side of a relevant target (but see^56^), which was not a requirement and was even discouraged in our paradigm.

Another possibility is that VOA-like activity, reflecting transient orienting to novel distractors, may only emerge in the very first few trials^10^. After this initial phase, the unfolding of habituation could rapidly suppress this orienting response, while continued habituation would further reduce any residual interference from the distractor throughout the experiment. If this is the case, averaging across entire blocks might obscure the early VOA ERP. While the absence of a VOA might be interpreted as a lack of attentional capture, the behavioral data suggest otherwise: RTs were reliably modulated by distractor presence, particularly in early blocks, indicating a measurable interference effect. Thus, future research is needed to more precisely determine how VOA-like components relate to distractor suppression and habituation dynamics.

Mechanisms involved in handling distractors have been often related to neural circuits in the frontal and parietal cortex, with the middle frontal gyrus playing a special role^50,57–60^. Consistent with this, our findings also indicate the involvement of frontal areas, as suggested by the source localization of reactive components of habituation, pointing to regions of the frontal and mid-frontal cortex, and extending to the middle frontal gyrus (Fig. 2D). The increased source activity in these areas may reflect the strengthening of top-down attentional inhibitory signals during habituation, acting as a filter to attenuate further attentional engagement and sensory processing of the distractor. This interpretation is supported by the concurrent decreases in source activity observed over parietal and occipital regions, which suggests a reduction in further distractor processing as frontal filters become more active, accompanied by reduced engagement of parietal sources of attentional control^25^.

These findings suggest a transition between neural networks during habituation, with occipital and parietal areas gradually decreasing their responsiveness to the distractor, disengaging sensory processing and attention, while frontal and mid-frontal regions become more active as habituation unfolds. This latter aspect likely reflects the activation of stereotyped automatic suppression mechanisms, possibly orchestrated by the middle frontal gyrus, which facilitate the rapid reallocation of attentional resources away from the distractor or prevents attention from being allocated to the distractor in the first place.

In addition to ERP effects, we observed a global increase in α power throughout the experiment. As noted earlier, this increase may not directly reflect habituation mechanisms, as α activity parameters can vary with time on task, reflecting nonspecific effects like fatigue or task automaticity^36,37^. To isolate the effects of pre-stimulus α power on RT in trials with and without distractors, we applied residualization techniques^61^ and found dissociable effects: higher α power around the target onset correlated with slower RTs in trials without a distractor, while higher α power around the expected distractor onset correlated with faster RTs in trials with a distractor (see Fig. 4A,B). This suggests that stimuli occurring during states of high α power require more time to be recognized (e.g., the target) or interfere less with the main task (e.g., the distractor).

These findings are consistent with the well-established inhibitory role of α oscillations in attention and neural processing^25,27,30–32,62–68^ and support the view of α oscillations acting as top-down gating mechanisms^25,30–32^. In line with these frameworks, pre-stimulus α power effects were source-localized in occipital and more anterior regions including parietal and mid-frontal areas, indicating a distributed network in which top-down signals from parieto-frontal attention networks may exert inhibitory control over distractor processing in sensory areas^25,32,69^. Importantly, effects of α power on distractor-present trials were not evident during the entire pre-stimulus window, but exhibited a clear temporal alignment around the time of the potential occurrence of a distractor (Fig. 4A), suggesting proactive mechanisms that anticipate the temporal onset of the stimulus^70,71^.

To understand the relationship between α power effects and habituation, we further analyzed α power effects separately for the first (block 1–2) and second (block 3–4) halves of the experiment, which correspond to the two stages where the RT cost due to distractors significantly differed because of habituation (Fig. 1C). We found that the effects of α power on trials with distractors were most pronounced in the second part of the experiment (blocks 3–4. Figure 4D). We hypothesize that this may reflect the establishment and consolidation of habituation mechanisms for the automatic inhibition of distractors or of the related response. Initially, distractors may capture attention, causing interference with the main task (block 1–2), but over subsequent blocks (3–4), they become integrated into an internal model of the irrelevant stimulation, leading to an attenuated attentional response. Our results suggest that α activity may play a key role in this process.

Beyond its traditional role as an inhibitory rhythm, indeed, our findings suggest that α activity may be causally involved in the instantiation and consolidation of internal models, or negative templates^18^, reflecting neural correlates of anticipatory stages of habituation^12^. These templates, critical for distractor suppression and habituation, incorporate both the expected characteristics of a distractor and time-based expectations about its likely occurrence. This view, supported by previous behavioral research^21^ and the temporal tuning of α power observed in our study, resonates with classic habituation models, where previous stimuli are integrated into an internal model that is constantly updated, and responses to stimuli that match predictions of the model are inhibited^12,33^.

Following the ‘stimulus-model comparator’ theory^12^, the observed anticipatory effects of α power may thus reflect the forecasts of internal ‘negative’ models for distractor inhibition, involved in the anticipatory stages of habituation. This possibility appears in line with recent research suggesting α traveling waves as potential mechanisms for top-down predictions within the framework of predictive coding^72^ and with behavioral evidence showing that distractors may be tagged with negative traces that cause subsequent perceptual decisions to be repelled away by their features^17^.

Traditionally, reactive and proactive components of distractor suppression have been viewed as distinct mechanisms^29^. However, in agreement with and as postulated by Sokolov’s model, our findings suggest that these two stages, despite being associated with different EEG correlates^73^, may work in concert within the context of habituation. Anticipatory α may align current sensory input with predictions of an internal distractor model, initiating local inhibition in sensory circuits and downregulating the transmission of distractor signals^25^. Concurrently, habituation reinforces the functioning of filters that reactively suppress attentional processing and counteract residual distraction. The observation that, at the single-trial level, pre-stimulus alpha power modulations also predicted the magnitude of the ERP component associated with habituation suggests that these two mechanisms may be part of the same process rather than independent components^74^. It is possible that proactive mechanisms matching the stimulus to the forecast of an inhibitory model also trigger reactive mechanisms to counteract distraction depending on the match between the stimulus and the model^12,13^. This adaptive mechanism would be beneficial when encountering a novel stimulus, allowing for the flexible allocation of attention to novel events that do not conform to the model’s predictions^75^. Future studies may investigate how these components behave when violating the model’s predictions, such as removing the distractor for a block of trials or unexpectedly varying its characteristics and temporal onset.

As mentioned throughout the manuscript, our study presents several potential limitations due to the use of a simple habituation paradigm in EEG. First, the absence of a contralateral ‘reference’ neutral stimulus limited our ability to perform classic lateralized ERP analyses and subtraction methods. Such approaches might have been useful to assess a more direct link—if present—to standard lateralized ERP components frequently reported in tasks involving multielement displays. This would have been particularly relevant for interpreting our findings in relation to classic ERP components associated with distractor suppression, as opposed to broader effects related to stimulus processing and attentional re-engagement. However, following previous studies^76,77^, we present an analysis based on lateral electrodes in the Supplementary Material, showing changes in both contralateral and ipsilateral ERPs over the course of the experiment. These results are consistent with the broad scalp effects reported in our main analysis (Fig. 2A).

Second, our experimental design and analytical approach faced challenges related to disentangling the confounding effects of time-on-task, which could have influenced several variables of interest. To address this, we employed a dedicated statistical approach to account for and residualize time-on-task effects in all relevant analyses. Nonetheless, future studies with more targeted designs will be necessary to better control for and directly assess these potential confounds. Additionally, the close temporal proximity of distractors and targets in our paradigm precluded a detailed investigation of the effects of distractor habituation on target-related ERPs, as the latter were likely contaminated by distractor ERP activity and its modulation over time. Finally, while we interpret our findings within the well-established framework of habituation and its neural correlates in attentional processes^10,11,16,18,20,21,33,41^, our current design primarily assessed the basic manifestation of habituation—namely, the reduction in RT cost. Additional investigations will be necessary to test other fundamental and more specific characteristics of habituation^2,78,79^, in order to provide more robust support for our conclusions.

In sum, our study offers initial evidence suggesting that habituation to visual distractors may be associated with changes in EEG activity, occurring both in reaction to and in anticipation of repeated distractors. These findings point to the involvement of a multi-stage chain of neural processes supporting habituation, consistent with the predictions of an internal negative model that supports inhibition and reactive suppression of repetitive irrelevant stimuli.

## Methods

### Participants

Twenty participants, students recruited from the University of Fribourg (Fribourg, Switzerland), including 3 males and one left-handed participant, with a mean age of 23 (SD = 3.5), participated in the experiment in exchange for course credits. To ensure normal or corrected-to-normal vision, participants underwent testing with the Freiburg Acuity test, requiring a minimum value of 1 with both eyes open for continued participation^80^.

All participants provided written informed consent before the experiment. The experiment was carried out in compliance with the Declaration of Helsinki and with the approval of the regional ethics board (CER-VD, Protocol Nr. 2016-00060). One participant was excluded due to technical issues during the EEG recordings resulting in a substantial portion of missing EEG data. A second participant was excluded due to poor performance (more than 25% of errors). The data from 18 participants were used for the analysis.

### Apparatus

Participants were seated in a dark and electrically shielded room, wearing a 128-channel BioSemi Active Two cap and electrodes (Biosemi, Amsterdam, The Netherlands). Task stimuli, created using Psychopy^81^, were presented on a VIEWPixx/3D display system (1920 × 1080 pixels, refresh rate of 100 Hz), from VPixx technologies. Behavioral responses were collected using a ResponsePixx response box (VPixx technologies).

### Stimuli and procedure

Figure 1A shows an example of the sequence of events on one trial. Each trial began with a red central fixation point presented for 700 ms. On distractor-present trials (40% of the total trials), a distractor appeared in the lower left or lower right quadrant of the display, 6° visual angle below the fixation point, and + /− 10° left or right of center for 100 ms, while the central fixation point was maintained. On distractor-absent trials, no distractor was presented, and the fixation point was maintained. Following this, a target was presented for 100 ms at the center of the screen. The interval between the fixation point and the target was always 700 ms. Thus, when a distractor was present, it appeared within this interval before the target, with the precise timing determined by a randomly selected target-distractor SOA, ranging from 50 to 200 ms in 25 ms increments.

Distractor stimuli consisted of full contrast horizontal black and white checkerboards displayed within a circular frame. Target stimuli were five-by-five grids of black and white dots. Participants were instructed to report whether there was a majority of black or white dots in the target grid by pressing the corresponding button on a ResponsePixx response box (VPixx technologies). Incorrect responses were followed by auditory feedback.

The experiment comprised 4 blocks of 150 trials each, with a brief practice session preceding the main experiment. The entire experiment lasted approximately 30 min and was one component of a battery of three visual tests, two of which have been published^82^.

### EEG preprocessing

To ensure the quality of EEG recordings, electrode offset values between active electrodes and a reference (Common Mode Sense – Driven Right Leg, CMS-DRL feedback loop) were verified to be within the range of − 20–20 mV.

EEG data were preprocessed using the EEGLAB toolbox in MATLAB^83^, following the procedure outlined in^82^. Briefly, preprocessing included down-sampling to 250 Hz, detrending, line noise removal with spectral interpolation, epoching, removal of bad trials and channels, independent components analysis, and manual removal of bad components (5.6 ± 4 ICA removed on average across subjects). EEG epochs were rejected through visual inspection, focusing on signal drops, motion artifacts, and large drifts in the data, following a procedure validated in previous studies^82^. Epochs containing eye blinks at the onset of target and distractor stimuli were excluded; remaining eye-related artifacts were addressed using ICA. After cleaning, less than 3% of epochs were removed on average. The proportion of rejected epochs was comparable between distractor-present and distractor-absent conditions (absent: 2.19% ± 2.40%; present: 2.87% ± 3.56%).

At the end of preprocessing, bad channels were interpolated from the data (less than 15% on average), and the data were referenced to the average of all (good) electrodes. Additional details can be found in^82^. Epochs were defined as segments time-locked to the distractor or the target onset, as specified for each analysis in the Results section. In the analysis of ERP, data were also lowpass filtered at 20 Hz and baseline corrected (baseline computed from − 200 to 0 ms before the distractor onset). In time–frequency analyses, data were down-sampled to 100 Hz.

### Behavioral analysis of habituation

We assessed habituation to visual distractors following the approach validated in previous work^11^. Trials behavioral errors or RT faster than 150 and slower than 1000 ms were excluded from the main analysis (9 ± 3% on average). Overall, the proportion of excluded trials—including both EEG epochs and behavioral outliers—was slightly higher in the distractor-present condition compared to the distractor-absent condition (absent: 11.02% ± 3.82%; present: 13.06% ± 5.57%), primarily due to a modest increase in error rate. However, this difference was negligible (~ 2%) and is unlikely to have significantly affected the results of the analysis presented below.

The analysis focused on changes in the RT cost due to a distractor over time, achieved by categorizing trials into the four experiment blocks and computing a metric of distractor interference (the positive difference between RT in distractor-present and distractor-absent trials, where a higher value indicates stronger interference) for each block.

Repeated measures analysis of variance (ANOVA) was used to assess changes in RTs over the experiment and as a function of the distractor presence (with factors Distractor and Block), followed by post hoc t-test analysis. The Bonferroni correction was employed for multiple comparisons. Effect sizes (Cohen’s *d’*) and 95% confidence intervals, corrected for within-subjects repeated-measures designs^84^, were reported in the analyses and plots, respectively. Participants performed the task with an average percentage of correct responses of 92% + /− 3% in distractor absent trials and 91% + /− 4% in distractor present trials, with no significant difference in accuracy in any of the blocks (all *p values* > 0.05 after correction for multiple comparisons).

### ERP analysis

To assess the impact of habituation on the distractor-evoked response, we employed a series of log-linear regression models to predict changes in ERP amplitude (z-scored) based on trial number (using log-transformed trial number to account for the non-linear function of habituation^10^). This analysis was performed for each channel and at each time point, initially distinguishing between distractor sides and running models at the single-subject level.

To test the significance of the regression coefficients at the group level, which reflect ERP changes associated with increasing trial numbers, we conducted paired t-tests. These tests evaluated the null hypothesis H0 that β = 0, comparing the coefficients across subjects to zero. Results were corrected for multiple comparisons using a false discovery rate (FDR) correction with a 5% threshold. Figure 2A shows results at both uncorrected (α = 0.05, opaque regions) and FDR-corrected levels (areas outlined in black). The topography was then plotted within the time window exhibiting the largest effect size (160–192 ms), determined as the interval where the sum of absolute significant T-values (Σ(|T-values|)) surpassed the 95th quantile (after correction).

The results for both left and right sides demonstrated similar non-lateralized patterns of significant effects emerging after the initial typical visual ERP (e.g., after ~ 100 ms). Consequently, for subsequent analyses, we collapsed the side factor. To further examine the effect at the ERP level, we identified electrodes with the largest T-values in both negative and positive directions from the combined topography of left and right distractors. Subsequently, we plotted the ERP to visually compare average waveforms in the first and last blocks of the trials.

### Characterizing the relationship between ERP and RT

To examine the effect of distractor ERP on RT, we applied the following linear mixed model:

1 $$log \left(RT\right) ={\beta }_{0}+{\beta }_{1}ERP+{\beta }_{2}log \left(trial\right)+\left(1|ID\right).$$

We predicted single trial log-transformed RTs $log \left(RT\right)$ using ERP amplitudes averaged within the 160–192 ms time window, focusing on a representative occipital electrode (see “Results”, “ERP correlates of habituation”). Both ERP amplitude ${\beta }_{1}ERP$ and log-transformed trial number ${\beta }_{2}log \left(trial\right)$ were included as fixed and random effects, with participant ID $1|ID$ as an additional random effect. This approach allowed us to assess how the observed ERP modulations over the course of the experiment related to behavioral responses, specifically RTs in distractor-present trials, while controlling for potential confounding effects of trial number such as time-on-task.

### Pre-stimulus spectral analysis

To investigate changes in pre-stimulus EEG activity throughout the experiment, we analyzed the power spectral density (PSD) within the 4–40 Hz frequency range using a 1024-point fast Fourier transform (FFT) implemented in MATLAB (R2020b). The PSD was computed for each trial within a distractor-locked time window spanning from − 700 to 100 ms.

Our analysis involved two levels. First, at the individual participant level, we employed a linear model to predict single-trial PSD (in decibels) with an intercept and a slope associated with the log-transformed trial number effect. Second, at the group level, we assessed the significance of the slope by conducting a two-tailed t-test against 0, with a significance threshold of α_*crit*_ = 0.05. We applied a FDR correction with a 5% threshold to address multiple comparisons.

To identify the frequencies exhibiting the strongest effect of trial number, we summed all significant T-values (Σ(|T-values|)) across electrodes. We selected the frequency range showing values surpassing the 95th quantile (after correction), revealing a clear peak in the α range between 9 and 11 Hz (9.27–10.74 Hz).

### Time–frequency analysis

To inspect the temporal dynamics of EEG oscillations with respect to the expected onset of a distractor (from − 200 to − 50 ms before the target), we calculated the wavelet power for each trial in the 4–40 Hz frequency band using the Morlet wavelet decomposition (number of cycles = 6, fixed for all frequencies). We focused on trials without a distractor and time-locked the data to the target onset (from − 1000 to 300 ms). This approach allowed us to assess the overall change in EEG rhythms around the time of the distractor, even in trials where the distractor was absent. Subsequently, the time–frequency power was averaged across electrodes and compared between the first half (blocks 1 and 2) and the second half (blocks 3 and 4) of trials. The reported results are presented as Cohen’s *d’* for this difference (Fig. 3B).

### Characterizing the relationship between pre-stimulus α and habituation

We analyzed the relationship between pre-stimulus α oscillations and distractor interference by time-locking EEG data to the target onset and estimating the time course of α power using the Morlet wavelet transform within a window from − 712 to 96 ms around the target onset. Specifically, we assessed whether α power, within the previously determined frequency range (9–11 Hz), could predict variations in RTs to the target, taking into account the presence or absence of a distractor.

Behavioral analysis of habituation indicated that RTs in the primary task changed with the interaction between distractor presence and the increasing number of trials (blocks). Pre-stimulus α oscillations were also significantly influenced by the growing number of trials. To isolate the direct impact of ongoing α oscillations on RTs, beyond the common effects of trials, we adopted a variable residualization approach. Single-trial log-transformed RT ($log \left(RT\right)$), aggregated from all participants, were initially fitted to the following linear mixed model:

2 $$log \left(RT\right) ={\beta }_{0}+{\beta }_{1}D+{\beta }_{2}log \left(trial\right) +{\beta }_{3}Dlog \left(trial\right) +\left(1|ID\right).$$

Here, ${\beta }_{0}$ is the intercept, ${\beta }_{1}D$ is the effect of the distractor absence or presence (with D coded categorically), ${\beta }_{2}log \left(trial\right)$ is the effect of the increasing number of trials, and ${\beta }_{3}Dlog \left(trial\right)$ is the interaction between trials and distractor conditions. Participant ID was introduced as a random effect. Residuals from this model (${RES}_{log \left(RT\right)}$) represented RT fluctuations not explained by the distractor’s presence, trial numbers, or their interaction.

Similarly, we modeled α power, averaged over the entire scalp and z-scored across trials at each time point $t$ of the pre-target interval, as a function of trial number:

3 $${\alpha }_{t}={\beta }_{0}+{\beta }_{1}log \left(trial\right) +\left(1|ID\right).$$

Residuals from this second model (${RES}_{{\alpha }_{t}}$) provided a predictor variable expressing fluctuations in α activity across trials not solely due to trial number effects.

The relationship between α and RT was assessed using a sliding linear mixed model:

4 $${RES}_{log \left({RT}_{t}\right) }={\beta }_{0}+{\beta }_{1}{RES}_{{\alpha }_{t}}+{{\beta }_{2}RES}_{{\alpha }_{t}}:D+\left(1|ID\right).$$

Here, the residualized log-transformed RT was modeled with coefficients ${\beta }_{0}$, ${\beta }_{1}$, and ${\beta }_{2}$ accounting for the intercept, time-varying effects on RT due to changes in ${RES}_{{\alpha }_{t}}$ without a distractor, and effects of α power specific to RT in the presence of a distractor, respectively. The model was performed for each time point, and participant ID was introduced as a random effect. Significance of the coefficients of interest (${\beta }_{1}{RES}_{{\alpha }_{t}}$; ${{\beta }_{2}RES}_{{\alpha }_{t}}:D$) was assessed using a false discovery rate correction (FDR) with a 5% threshold to address multiple comparisons when testing across $t$ (Fig. 4A). The results are represented in a plot showing the time course of the estimated coefficients of interest with the related standard error (Fig. 4A). Additionally, we also plotted the significant interaction resulting from eq. (4) by representing residualized RT in distractor absent and present trials as a function of residualized α power quantiles (4 quantiles, in increasing order, Fig. 4D), facilitating a more immediate interpretation of the interaction. Importantly, we verified that all modeling results were consistent with non-residualized models that included log(trial) and distractor effects, as well as models with a more complex random effects structure. Given that the effects of time-on-task (trial) and distractor condition on RT were relatively static, we chose to focus on the pre-stimulus time dynamics of α power using this residualized approach.

To determine whether the effects identified by the mixed linear model were consistent throughout the entire experiment or specific to distinct habituation stages, we ran model (4) separately for the first (blocks 1–2) and second (blocks 3–4) halves of the experiment. These two stages correspond to when the RT cost due to the distractor was still significant (blocks 1–2) and when it had significantly decreased (blocks 3–4), reflecting habituation. In this analysis, we focused on the average ${RES}_{\alpha }$ effect within the time window exhibiting a significant interaction ${RES}_{{\alpha }_{t}}:D$, as identified in (3). The results are represented as the coefficient ${\beta }_{2}$ along with 95% confidence intervals for the interaction ${RES}_{\alpha }:D$ estimated separately for each half of the experiment (Fig. 4C).

To explore the EEG topography associated with the interaction between α power and distractor presence, we applied the same mixed model (4) to individual electrodes after averaging α power within the significant time window of the ${RES}_{{\alpha }_{t}}:D$ interaction. The resulting T-values of the estimated ${\beta }_{2}$ coefficients were then visualized as EEG topography, with electrodes exhibiting effects larger than the 95th quantile highlighted (Fig. 4B).

To assess the relationship between pre-stimulus α power modulations and changes in post-stimulus topographies in response to the distractor, we ran an additional linear model. In this model, we predicted the ERP, averaged within the significant time window identified in the *ERP analysis,* and for each of the two electrodes exhibiting peak effects (A21, D1, see Fig. 2B). The predictor was based on single-trial α power values, averaged within the electrodes and time window that demonstrated the maximum interaction effects ${RES}_{{\alpha }_{t}}:D$ on RT (Fig. 4A,B). We removed the log(trial) effect from both variables during this analysis.

### EEG source imaging

In the localization of habituation effects, we conducted source imaging using individual head models and electrode locations. The head models were generated from individual structural magnetic resonance (MR) data collected from the same subjects and spatially aligned to the individually digitized electrode locations. Detailed information on the acquisition and preprocessing of MR data can be found in^82^. Anatomical MR data were segmented to obtain border surfaces between the scalp, skull, and brain, and volume conduction models were obtained with the boundary element method using OpenMEEG^85^. Source locations were derived from a template grid based on the Montreal Neurological Institute and Hospital (MNI) template anatomical MRI (a grid of equivalent current dipoles with 6 mm regular spacing, constrained within cortical gray matter, with 4579 source points inside). Individual anatomical images were warped to the template, and the inverse operator was applied to the template grid to derive individual grids^31,32^. Forward models were then obtained assuming fixed dipole orientations.

For the localization of habituation effects in post-stimulus evoked responses, we used the linearly constrained minimum variance (LCMV) beamformer (lambda = 10%, weights normalization: nai;^86^. The data covariance matrix was estimated within a post-stimulus window from 50 to 500 ms after the onset of the distractor. The time window of interest (160 to 192 ms) was selected based on a data-driven analysis following ERP results. Source activation results were averaged over time and analyzed using a linear model, similar to the ERP analysis. For this analysis, EEG data from the four blocks of trials were divided into a total of 12 sub-blocks to enhance the signal-to-noise ratio and reduce computational costs compared to single-trial estimates. Source activity was estimated on the average ERPs of each sub-block using a common covariance and spatial filter derived from all trials. Sub-block source results (averaged absolute source activity in the time window of interest) were then subjected to a linear model. This involved fitting, for each subject and source point (N = 4579), a model with an intercept and the variable log(sub-block) as the main predictors. To further improve signal-to-noise, we smoothed source activity values at each source point over the 12 sub-blocks via linear moving average (window size = 3 sub-blocks). We collected T-values associated with the slope—i.e., the variation in source activity across trial sub-blocks— and the effect size at each source point was estimated at the group level, using Cohen’s *d’* (as implemented in *ft_statfun_cohensd* in fieldtrip). The results are plotted after thresholding for a minimum effect size of Cohen’s *d’* = 0.2 (small effect size) in both negative and positive directions, where positive values indicate increases in source activity across sub-blocks and negative values indicate decreases. For graphical representation, the results were interpolated, projected onto a cortical surface model provided as a template in Fieldtrip (surface_white_both.mat), and smoothed with 5 voxels full-width at half maximum–FWHM Gaussian kernel. Smoothing and visualization were performed using *bspmview* in MATLAB (http://www.bobspunt.com/software/bspmview).

For the localization of pre-stimulus power effects in the α band (selected in a data-driven way, 9–11 Hz, see “Pre-stimulus spectral analysis”), we employed Dynamic Imaging of Coherent Sources (DICS; as implemented in fieldtrip v.20210614, with lambda = 10%), a frequency domain beamforming approach^87^. This analysis focused on localizing coherent sources of α power that could be associated with the interaction between distractor condition and α power effects on RT found in the scalp (see “Characterizing the relationship between pre-stimulus α and habituation”).

To this end, we extracted source α power in a time window derived from scalp analysis (from − 142 to 48 ms), separately for distractor present and absent conditions. We further categorized trials within each distractor condition based on slow or fast RT (RT faster than the 25th or slower than the 75th quantile). This categorization was applied to RT residualized from the effects of condition, trials, and their interaction (eq. [4]), following the logic described in “Characterizing the relationship between pre-stimulus α and habituation”.

Similar to the LCMV analysis, coherent sources for each condition were estimated using a common spatial filter derived from all trials. For each source point, data from all subjects were then subjected to a two-way repeated measures ANOVA with factors Distractor (absent vs. present) and RT bin (slow vs. fast). The resulting F statistic of the interaction term (Distractor * RT bin) was then thresholded with a logical operator imposing the fulfillment of three conditions: (1) a significant interaction (*p* < 0.05 unc.), (2) larger source α power in the fast compared to the slow RT bin in the Distractor present condition, and (3) an opposite or smaller trend of source α power over RT bins in the Distractor absent condition.

These criteria allowed retaining F values at source points where the relationship between source α power, Distractor, and RT resembled the one found in scalp analysis (e.g., Fig. 4D). The resulting map of F statistics was then projected onto a cortical surface model provided as a template in Fieldtrip (surface_white_both.mat), smoothed, and plotted following the same approach as in the LCMV analysis. All source results were additionally assessed using a template, rather than individual head models, providing largely consistent results (see Supplementary Material, Figure S3).

## Supplementary Information

Supplementary Information.

## Data Availability

The data used in this study are available from the corresponding author upon request.
