# Supplementary material for: Anticipatory and reactive mechanisms of habituation to visual distractors
Source: Sci Rep. 2025 Jul 2;15:22953. doi: 10.1038/s41598-025-04082-5 (PMC12218333; doi:10.1038/s41598-025-04082-5)
Supplement: Supplementary file 1 — Supplementary Information. [file 41598_2025_4082_MOESM1_ESM.docx]

# Supplementary Material


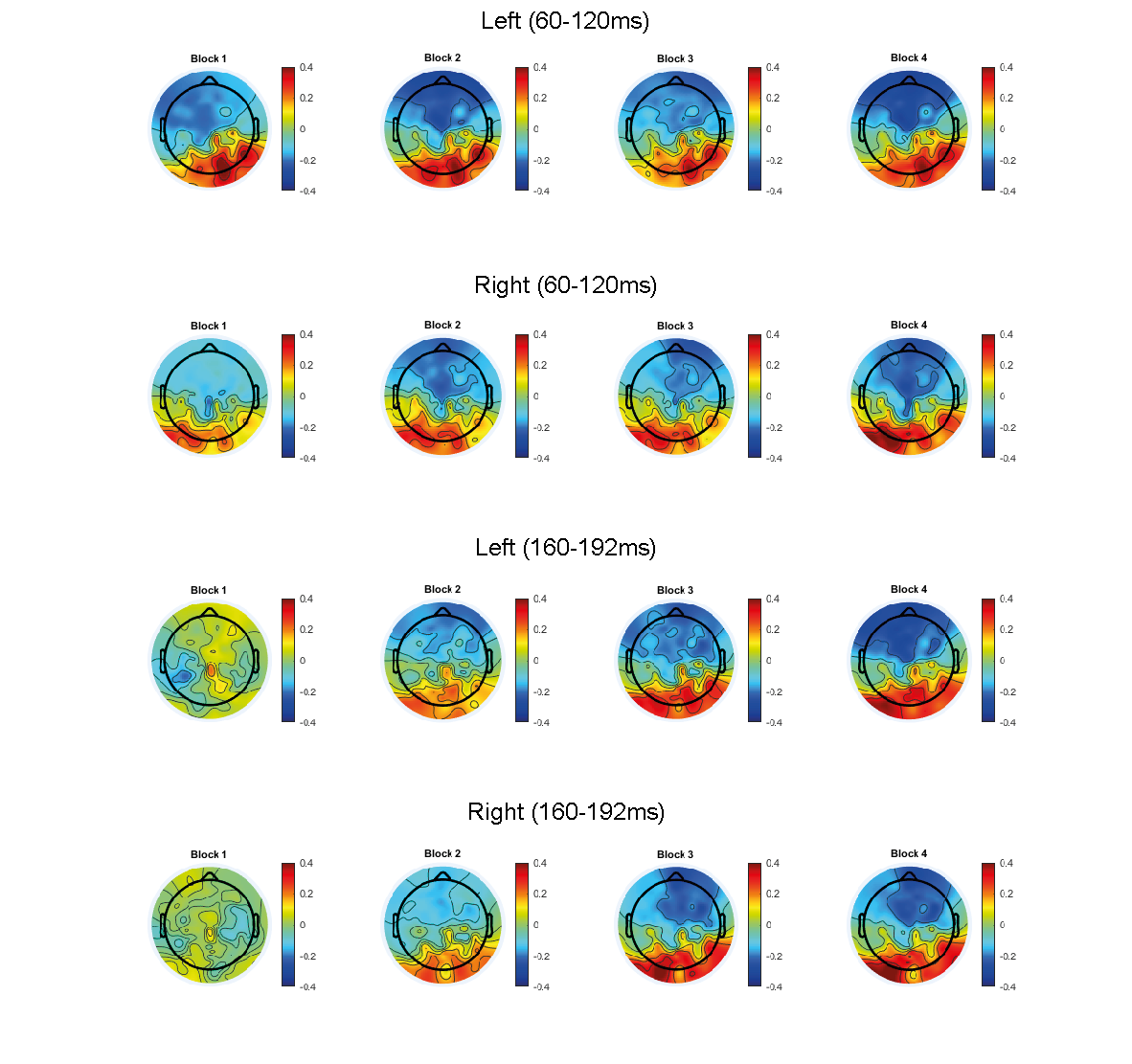


**Figure S1**. ERP topographies elicited by the peripheral distractor, averaged across participants, between early (60-120 ms) and late (160-192 ms, aligning with the time window of the habituation effect) time windows across blocks of trials (1 to 4). In the early time window, responses exhibit lateralized topographies, with positive deflections at occipital electrodes contralateral to the distractors. These early responses remain stable across blocks (first two rows). In contrast, during the late time window, ERP responses are no longer lateralized, and there is a marked decrease in overall amplitude across blocks (last two rows). The late scalp modulations observed align with distributed changes across the scalp, characterized by increased positive polarity over occipital regions and negative polarity over frontal regions. These findings are consistent with the modeling approach and the results shown in Figure 2 of the main text.


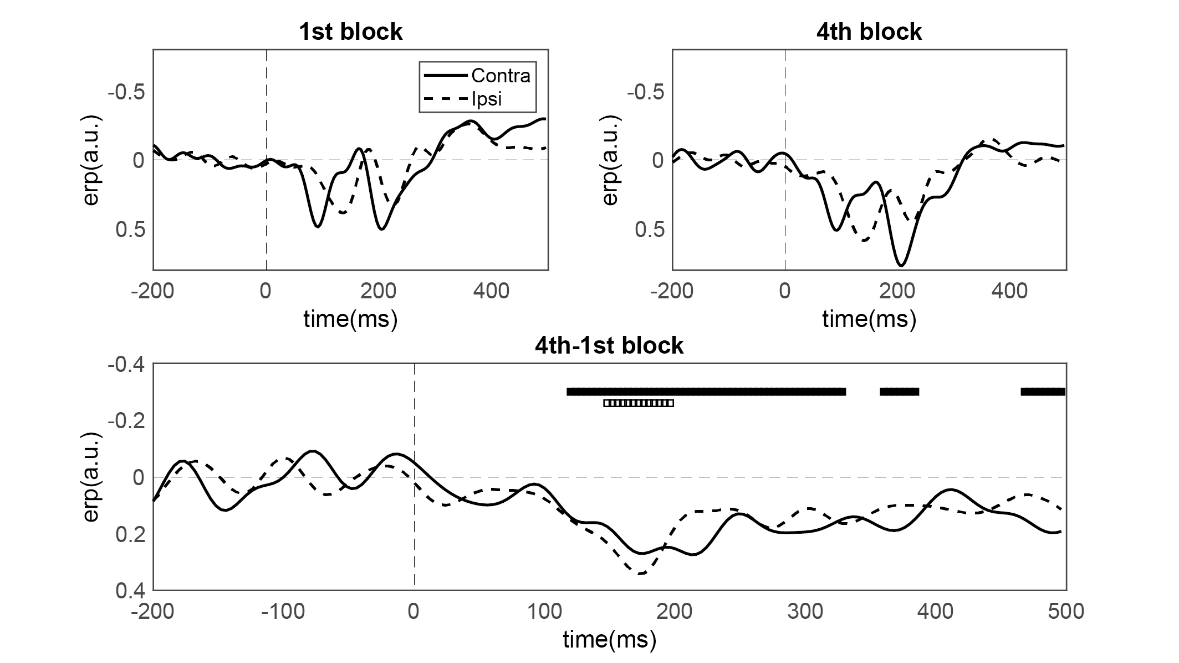


**Figure S2**. Contralateral and Ipsilateral ERPs at PO7/PO8 (collapsed across sides, as in McDonald et al., 2022). The top two panels display the contralateral (solid black line) and ipsilateral (dashed line) ERPs relative to the visual onset distractor, averaged across participants. The left panel represents the first block, and the right panel represents the last block. The ipsilateral ERP exhibits delayed main deflections compared to the contralateral ERP, consistent with findings from McDonald et al. (2022). The bottom panel shows the difference between the last and first blocks for both contralateral (solid black line) and ipsilateral (dashed line) ERPs. Both contralateral and ipsilateral ERPs demonstrate larger amplitudes in a time window starting after 100 ms, with significant time points (after FDR correction) indicated by horizontal filled squares (contralateral) and open squares (ipsilateral). No modulation specific to the ipsilateral component, such as the visual onset activity (VOA), was observed. For comparison with conventional ERP plots (e.g., McDonald et al., 2022), the y-axis sign direction has been reversed.


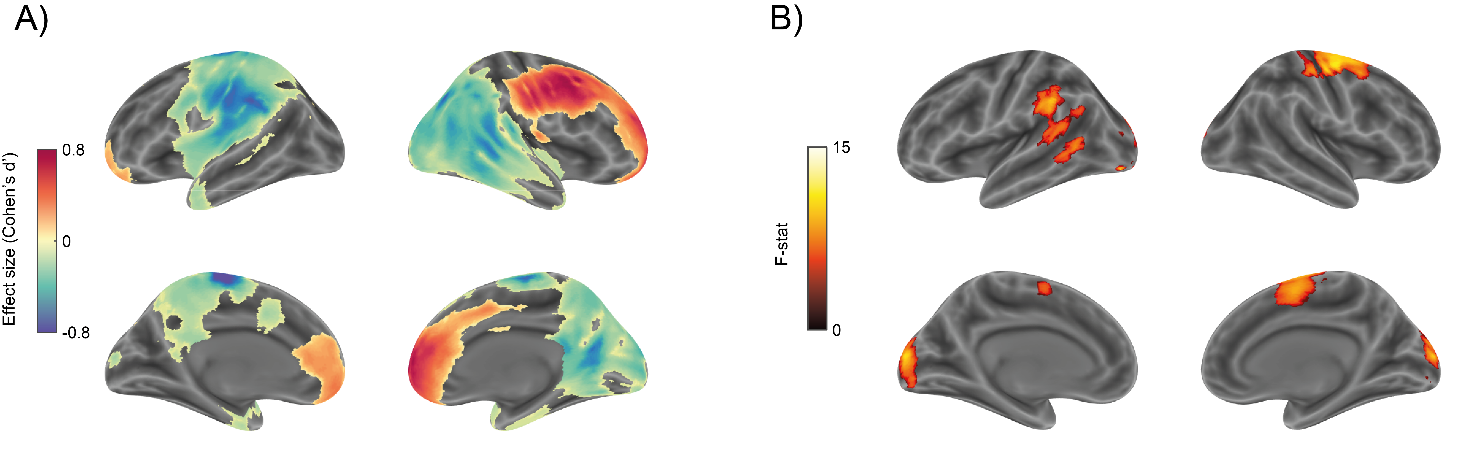


**Figure S3**. Source localization results using a template head model (see Methods: EEG source imaging). A) Source localization results of the effect of trials on post-stimulus ERP, using LCMV beamforming (see Figure 2D in the main text for the results with individual head models). B) Source localization results for the interaction effect in Figure 4E (see main text), using DICS beamforming.

**Supplementary Table 1**

| **ERP LMCV Individual** |  |  |  | **MNI Coordinates** | | |
| --- | --- | --- | --- | --- | --- | --- |
| **Contrast** | **Region Label** | **Extent** | **d'** | **x** | **y** | **z** |
| **Positive** | R Postcentral Gyrus | 4834 | 0.89 | 50 | -18 | 42 |
|  | R Middle Frontal Gyrus | 4834 | 0.50 | 28 | 14 | 54 |
|  | R Rolandic Operculum | 4834 | 0.50 | 44 | -22 | 22 |
|  | Location not in atlas | 6968 | 0.71 | 16 | 58 | -14 |
|  | R Superior Frontal Gyrus | 6968 | 0.43 | 22 | 52 | 18 |
|  | Location not in atlas | 6968 | 0.34 | 34 | 30 | 2 |
|  | L Middle Occipital Gyrus | 34 | 0.27 | -18 | -92 | 16 |
|  | L Middle Occipital Gyrus | 50 | 0.26 | -36 | -82 | 10 |
|  | L Middle Frontal Gyrus | 55 | 0.24 | -38 | 30 | 38 |
|  | R Caudate Nucleus | 93 | 0.24 | 12 | 8 | 14 |
| **Negative** | L Middle Frontal Gyrus | 50539 | -0.23 | -22 | 4 | 64 |
|  | R Fusiform Gyrus | 50539 | -0.24 | 26 | -40 | -8 |
|  | R Linual Gyrus | 50539 | -0.24 | 16 | -58 | -2 |
|  | R Fusiform Gyrus | 50539 | -0.80 | 20 | -52 | -8 |
|  | R Middle Temporal Gyrus | 50539 | -0.79 | 46 | -54 | 18 |
|  | R Inferior Parietal Lobule | 50539 | -0.74 | 42 | -54 | 50 |
|  | R Postcentral Gyrus | 1 | -0.21 | 50 | -34 | 58 |
|  | R Superior Medial Gyrus | 1 | -0.20 | 4 | 32 | 58 |
|  | R Rectal Gyrus | 6968 | 0.23 | 8 | 56 | -14 |
|  |  |  |  |  |  |  |
|  |  |  |  |  |  |  |
| **Frequency DICS Individual** |  |  |  | **MNI Coordinates** | | |
| **Contrast** | **Region Label** | **Extent** | **F-stat** | **x** | **y** | **z** |
| **Positive** | L Calcarine Gyrus | 4212 | 31.75 | 0 | -100 | 2 |
|  | L Superior Temporal Gyrus | 4212 | 9.94 | -60 | -54 | 26 |
|  | Location not in atlas | 4212 | 9.49 | -36 | -90 | -16 |
|  | R Paracentral Lobule | 8046 | 13.07 | 6 | -30 | 68 |
|  | L Posterior-Medial Frontal | 8046 | 10.72 | 0 | -10 | 62 |
|  | Location not in atlas | 8046 | 9.87 | 22 | -28 | 56 |
|  | R Cerebellum (Crus 1) | 432 | 6.18 | 36 | -72 | -22 |
|  | R Superior Frontal Gyrus | 162 | 5.76 | 18 | 24 | 56 |
|  | R Cerebellum (Crus 1) | 27 | 5.54 | 54 | -48 | -28 |
|  |  |  |  |  |  |  |
| **Note** |  |  |  |  |  |  |
| Table shows all local maxima separated by more than 20 mm. Regions were automatically labeled using the AnatomyToolbox atlas. x y and z =Montreal Neurological Institute (MNI) coordinates in the left-right anterior-posterior and inferior-superior dimensions respectively. | | | | | | |
